# Developing a risk management framework to improve public health outcomes by enumerating Salmonella in ground turkey – RETRACTION

**DOI:** 10.1017/S0950268822000954

**Published:** 2022-07-04

**Authors:** F. Sampedro, S. J. Wells, J. B. Bender, C. W. Hedberg

**Affiliations:** 1 Center for Animal Health and Food Safety, College of Veterinary Medicine, University of Minnesota, Saint Paul, USA; 2 Veterinary Population Medicine, College of Veterinary Medicine, University of Minnesota, Saint Paul, USA; 3 Environmental Health Sciences, School of Public Health, University of Minnesota, Saint Paul, USA

The Editors have chosen to retract this article on the grounds that there were significant procedural mistakes in the original paper, and a replacement paper is to be written. The errors are explained in detail in letters published in the journal.
